# Mechanisms and biomarker candidates in pterygium development

**DOI:** 10.5935/0004-2749.20200068

**Published:** 2020

**Authors:** Thiago Gonçalves dos Santos Martins, Thomaz Gonçalves dos Santos Martins

**Affiliations:** 1 Universidade Federal de São Paulo, São Paulo, SP, Brazil; 2 Ludwig Maximilians University, Munique, Alemanha; 3 University of Coimbra, Coimbra, Portugal; 4 Hospital da Piedade, Rio de Janeiro, RJ, Brazil

Dear Editor:

In response to the article titled “Mechanisms and biomarker candidates in pterygium development”, published in your esteemed journal, which is a well thought out and written paper, I would like to raise few points regarding this study.

Pterygium may have among its causes factors such as: changes in cholesterol metabolism, inflammation, viral infection, oncogenic proteins, lymphangiogenesis, and epithelial-mesenchymal cell transition^(1)^. We would like to add one more factor that may be related to the pathophysiology of pterygium.

Healthy conjunctival tissues may have Nod-like receptor pyrin3 (NLRP3). In pterygium, the NLRP3/caspase-1 pathway may be abnormally activated, accompanied by aberrant expression of IL-18 and IL-1β. There is a correlation between the number of fibroblasts and NLRP3. Activation of the NLRP3/caspase-1 pathway may be a cause of angiogenesis and fibroblast proliferation, which could induce pterygium recurrence. Nod-3 (NLRP3), caspase-1, IL-18 and IL-1β pyrodynamic receptors are common markers of pyroptosis, which is a form of programmed cell death that includes the release of inflammatory factors^(2-4)^.

Mitomycin C use in pterygium surgery reduces the recurrence rate, suppressing angiogenesis and fibroblast proliferation, inhibiting NLRP3/caspase-1 pathway activation and the expression of inflammatory factors such as TGF-β1, VEGF, and IL-6^(2-4)^.
